# Combining 3D Printing and Microfluidic Techniques: A Powerful Synergy for Nanomedicine

**DOI:** 10.3390/ph16010069

**Published:** 2023-01-01

**Authors:** Federica Sommonte, Nunzio Denora, Dimitrios A. Lamprou

**Affiliations:** 1 Department of Pharmacy-Pharmaceutical Sciences, University of Bari Aldo Moro, Orabona Street 4, 70125 Bari, Italy; 2 School of Pharmacy, Queen’s University Belfast, Belfast BT9 7BL, UK

**Keywords:** additive manufacturing, microfluidics, 3D printing, nanomedicine, nanoparticles, drug delivery, sustainability

## Abstract

Nanomedicine has grown tremendously in recent years as a responsive strategy to find novel therapies for treating challenging pathological conditions. As a result, there is an urgent need to develop novel formulations capable of providing adequate therapeutic treatment while overcoming the limitations of traditional protocols. Lately, microfluidic technology (MF) and additive manufacturing (AM) have both acquired popularity, bringing numerous benefits to a wide range of life science applications. There have been numerous benefits and drawbacks of MF and AM as distinct techniques, with case studies showing how the careful optimization of operational parameters enables them to overcome existing limitations. Therefore, the focus of this review was to highlight the potential of the synergy between MF and AM, emphasizing the significant benefits that this collaboration could entail. The combination of the techniques ensures the full customization of MF-based systems while remaining cost-effective and less time-consuming compared to classical approaches. Furthermore, MF and AM enable highly sustainable procedures suitable for industrial scale-out, leading to one of the most promising innovations of the near future.

## 1. Introduction

Nanomedicine (NM) has suddenly appeared as one of the most interesting hot topic issues in the research world. To comprehend the magnitude of this phenomenon, the multidisciplinary aspect of NM should be considered, as it embodies the goal of diagnosing, treating, and preventing diseases, as well as studying them in an integrated manner with respect to knowledge of the molecular tools of the human body [1]. The result is the tremendous impact and potential of NM in combining nanotechnology, biomedicine, biomaterials, and pharmaceutical sciences in order to solve classical medicine-related problems [2]. NM, and more specifically nanotechnology, has recently found a wide range of applications for the treatment of a variety of pathological conditions that would otherwise be challenging to treat satisfactorily [3]. Examining some of the diseases with the greatest mortality rates to date, e.g., infectious diseases and cancer, it is impossible not to deduce that there is a growing demand for suitable and efficient therapeutic treatments [4,5]. According to recent data, cancer is expected to cause 420 million cases by 2025, due to global demographic growth [6]. This massive amount of data compels the scientific community to consider potential therapies in addition to chemotherapy, which, while currently the most widely used treatment, is still far from a safe cure [6].

Recognizing and resolving these issues is the only way to ensure effective care and treatment for all patients. Novel therapeutic treatments involving the use of innovative nanoparticulate systems for the targeted administration of anti-cancer drugs [7,8], biological molecules, and cancer vaccines are being researched to achieve this goal [9]. Since cancer vaccines promote the formation of antibodies against tumor cells, the potential to drastically reduce tumor-associated mortality has been proven [10].

Furthermore, the COVID-19 pandemic situation is an intriguing example of how timely this issue is. This unexpected event prompted the production of much-needed vaccinations. Both BioNTECH/Pfizer and Moderna’s vaccines are mRNA strand-based, and use lipid nanoparticles (NPs) as carriers [11]. As a result of the introduction of protein- and nucleic acid-based therapeutic approaches, the scientific community has witnessed a revolution in the traditional drug concept, ushering in a new era in pharmacology. The new discoveries have made DNA, mRNA, siRNA, and protein tools a concrete and viable reality in clinical applications [12]. From a physiological standpoint, mRNA is produced during the translation of genes into proteins. Since the protein transduction systems are located in the cell cytosol, this translation does not necessitate the delivery of the strand into the cell nucleus. Unlike DNA-based strategies, there is no risk of the mRNA strand integrating into the host cell’s genome and causing mutagenesis when using innovative therapy [11,13]. Furthermore, mRNA strands can remain active for several days, allowing for the amplification of the pharmacological effect, which has commercial implications. Along with the benefits, there are some drawbacks to using mRNA that could be overcome, thanks to advances in NM and nanotechnology [13]. Indeed, mRNA has a short plasma half-life, allowing for quick inactivation by RNA-si, as well as potential immunogenicity, which might be used to boost the immunological impact of vaccines [11,13,14]. As a result, NM has taken the lead in biologically active molecules delivery research, laying the groundwork for the development of novel formulations distinct from those currently available [15].

As a necessary consequence, there have been considerable investigations of nanostructured materials as novel drug delivery systems (DDSs) based on their accomplishments in contrast to current therapeutic treatments. The tremendous progress in DDS research is paving the way for a novel therapeutic concept based on the use of nanovectors to precisely deliver the drug of interest to the disordered target sites, enhancing the pharmacological effects on their own while limiting systemic toxicity [16,17]. Furthermore, the dimensional properties of nanovectors should be suitable for overcoming biological barriers present in the human body, which severely limit the entry of substances into particularly sensitive areas [3,18]. It is essential that the specific nanosystems have the proper size range as it affects their ability to reach target tissue, their biodistribution, cellular uptake, and removal by clearance systems [18]. Thus, alternative strategies for producing NPs with adequate features are required because traditional production methods are incapable of ensuring rapid, inexpensive, high-reproducibility synthesis [19]. The main challenge remains the inability to control the size and polydispersity index (PDI) in bulk solutions due to a lack of rigid control over mixing and reaction times [20].

The microfluidic (MF) technique has firmly established itself in the NM field, demonstrating in recent years the undeniable benefits it provides over classical approaches. According to the literature [21,22], MF is a promising tool for producing safe, rapid, extremely reproducible, and reliable DDSs. Despite the undoubted advantages, there are still factors that prevent MF from permanently imposing itself as a production facility—among them the high cost of the devices and the difficulty in discovering and testing new geometries [23,24]. The goal of this critical review was to focus on the potential of combining the MF technique with a three-dimensional (3D) printing technique based on this evidence. The latter technique, which has become a popular area of study applied to personalized NM [25], is one of MF’s most important allies. The combination of the two could allow the issues associated with device customization to be overcome, leading to significant advancements in subsequent production and application [26]. Indeed, a degree of control could be achieved at every stage of the manufacturing process, from device design to printing to experimental application, making studies in this field significantly more efficient. As a result, while this is still an unexplored synergy, efforts have been made to highlight the most promising aspects capable of providing a true functional advantage to personalized experimental NM.

## 2. Method

**Figure 1:**
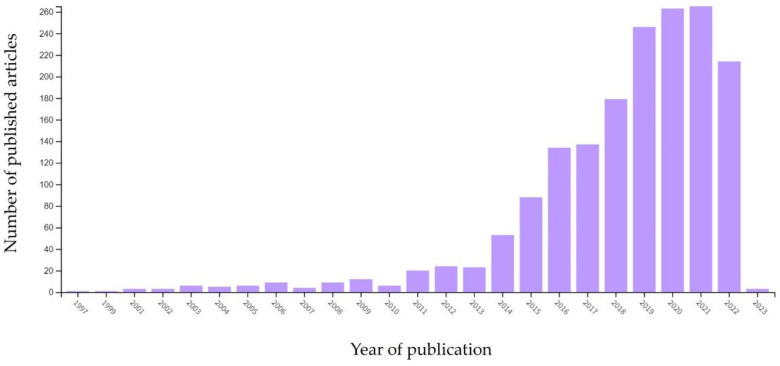
Growing trend of scientific publications using the keywords “3D printed microfluidic devices” according to Web of Science database [28].

The following data were collected after a careful review of currently available studies in the literature. The research was carried out by consulting different sources, including the Elsevier’s database Scopus [27] and Web of Science [28] using the search terms “3D printed microfluidic devices”, “drug delivery systems”, “nanomedicine”, and “microfluidic lab-on-a-chip”. Figure 1 depicts the growing trend of these topics according to the keywords “3D printed microfluidics”. It is worth noting that the number of scientific publications in these areas began to rise rapidly in 2014, and by 2018, there were three times as many as in 2014. Here, to focus the review on the most recent scientific findings, the last five years of scientific outcomes were reported.

## 3. Microfluidic Technique

**Figure 2:**
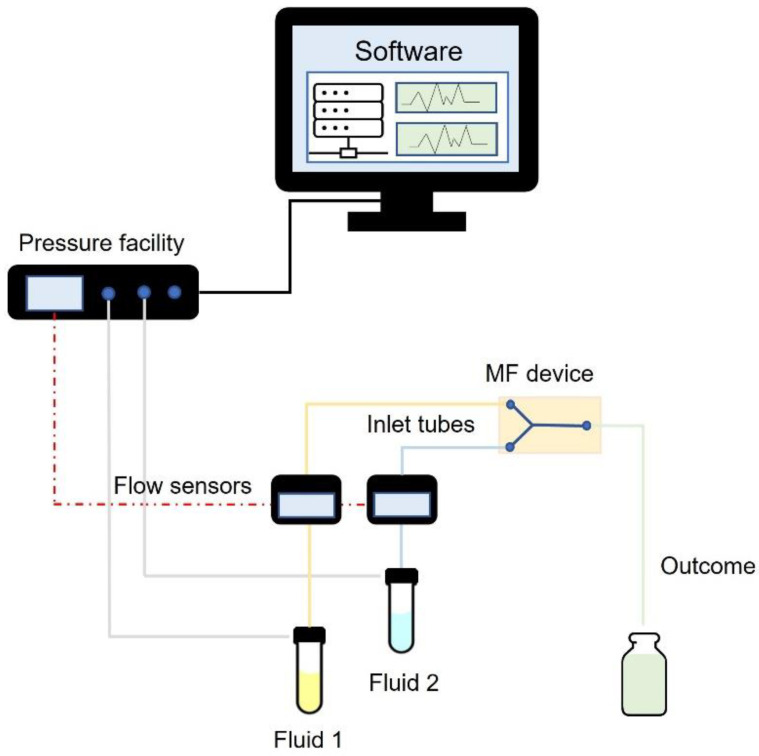
Schematic representation of MF-based setup.

MF is an attractive technology that involves the manipulation of microlitres of solutions inside devices with microchannels (<1000 μm) [29]. This feature allows to finely manage the fluid flow within the chambers, ensuring a high degree of reproducibility and scalability as the movement within the microchannels is time-to-time controlled [30]. The miniaturization of the system ensures the technique’s tunability due to the fluid-dynamic properties induced within an MF-based device (Figure 2). In fact, in contrast with bulk-based processes where fluids are turbulent and mainly driven by inertial forces, flows in a microscale system are laminar (low Reynold number) and rely primarily on viscous forces [30,31,32]. As a result of the considerable rise in surface-to-volume ratios in the MF system, diffusion processes drive mass transfer rather than convection forces, leading to a more controllable and reliable method [33,34]. The ability to explain this behaviour using mathematical equations allows each step of the process to be monitored, ensuring that the outcome is high quality [32]. The importance of this novel approach stems from the fact that it has a wide range of applications in various branches of scientific investigation [35], ranging from analytical purpose to the production of high-performance NPs [36,37,38], diagnostics [39] and cell analysis [40,41]. Among them, the concept of lab-on-a-chip (LOC) systems revolution has paved the way for many healthcare applications. LOCs are integrated microsystems in which the use of small MF chips ensures the integration of multiple processes on micro-scale platforms [42]. The great advantage of encompassing multiple laboratory functions in a single device has a key role in applied sciences, which reveals itself in less time-consuming processes, is more reliable in terms of quality assurance, and enables the reduction and/or elimination of a variety of unsuitable solvents [29].

It has been estimated that around 80% of pharmaceutical companies’ waste consists of exhausting solvents, the disposal of which is expensive and not eco-friendly. It is immediately necessary to achieve the most environmentally sustainable possible type of research, and MF, as an industrial scale-up technique, is currently an established focal point in this area [43].

### 3.1. Materials for MF Devices

One of the fundamental parameters to be taken into account when deciding to start MF manufacture involves the study of the chip to be used and the material from which it is made. The devices must have well-defined characteristics, including transparency; biocompatibility; and resistance to handling, temperature, pressure, and chemical compatibility with the used solvents [44]. Indeed, the miniaturisation of the channels that underlies the MF technique makes it essential to pay specific attention to the device’s constituting materials. Compared to macro-recipients, when considering reactions that take place on the micro-scale environment, it is necessary to contemplate all the variables that might affect the production process, including the wettability of the device’s material and the contact angle generated between the liquid phase and the micro-channel [44,45].

The following section encounters all materials that, historically and from an application standpoint, were most widely used in the fabrication of MF devices.

#### 3.1.1. Glass

The material with the best properties that can be adapted to the needs of MF is glass [37]. It is rigid, thermostable, resistant to all aggressive solvents, suitable for easy surface modification, chemically inert, and also suitable for biological substances [46]. Glass has excellent transparency and optical clarity and can be supplemented with accessory elements made of glass or other materials that can be added later [47]. Thus, glass could be a high-performance material, were it not for the high cost of MF chips. This is because although glass is low-cost on its own, the processes of machining and miniaturising the channels require expensive time-consuming procedures [48].

#### 3.1.2. Silicon

One of the first materials to be tested and used was silicon [49]. This material has excellent thermostability and chemical compatibility, is easily fabricated, and has an adaptable geometry design. It is highly flexible; however, this makes the incorporation of external accessory structures difficult. Furthermore, as it is not ultra-transparent, it is not suitable when optical detection in the visible or ultraviolet is required [44,46].

#### 3.1.3. Metals

Metals can be used for the manufacture of chips because they are cheap and easy to handle [50]. Chips made in this way withstand high working pressures and high temperatures, and are suitable for the use of substances that are incompatible with other materials. Aluminium, iron, and copper are the most-employed materials, although composites combining the properties of various metals are also widespread. They are also strong and easy to clean [44,50].

#### 3.1.4. Polymeric Materials

Polymer-based MF systems have gained wide acceptance in recent years, as they ensure reliable production under various temperature conditions, and have easily scalable properties. Furthermore, depending on the composition of the polymer, transparent or semi-transparent devices can be obtained that are applicable to all processes in which optical visibility is required [41,46]. The most commonly applied polymers include fluoropolymers, cyclo-olefin copolymers (COC)/cyclo-olefin polymers (COP), thiol-ene (TE) polymers, polydimethylsiloxane (PDMS) and polymethyl methacrylate (PMMA) are particularly relevant [44,51]. PDMS is cheap, easily mouldable, biocompatible, and therefore applicable in the biological field, very elastic and hydrophobic due to its chemical nature [52]. Its high porosity, however, makes it unsuitable for use with organic solvents, as the system could undergo a process of solvent absorption with consequent swelling [53]. PMMA is more resistant to organic solvents than PDMS, less porous to small molecules, is easy to handle being an amorphous thermoplastic material, is optically transparent, and has good mechanical properties [51].

#### 3.1.5. Paper

A more sustainable alternative to polymer chips is paper-based systems. These platforms are relatively inexpensive, environmentally friendly, easy to source, and can operate without support surfaces [54]. The great functionality of paper lies in its usage facility, in which the movement of fluids depends on their cohesive forces and the angle of contact with the cellulose material [55]. These devices have little mechanical resistance in high humidity conditions and cannot easily be applied to production processes. Therefore, they have been widely developed in rapid screening and diagnostic tests, exploiting colorimetric and electrochemical methods [44,54].

#### 3.1.6. Hydrogels

Hydrogels are polymeric structures that are extensively cross-linked and have a high degree of hydrophilicity [56,57]. Furthermore, are inexpensive and widely accessible, biocompatible, non-cytotoxic, biodegradable, and have the ability to modify cross-linking and pore width [57,58]. The use of hydrogels as starting materials for MF devices poses issues in terms of device integrity. Because of the structural resemblance of hydrogels to the extracellular matrix [59,60,61], applications have been developed in which this material is employed to simulate physiological tissues in so-called “biomicrofluidic” devices [62,63]. Table 1 highlights the prevalent ways of producing microfluidic devices based on the constituting materials that discussed in Section 3.

## 4. Three-Dimensional Printing

Current fabrication techniques for MF-based devices use typical cleanroom technology to avoid dust or other particulate deposition inside microchannels [26]. For example, glass devices are produced by wet etching, including procedures such as micro-machining, micro-milling, casting, hot embossing, and injection moulding [41,46]. These treatments result in resource-intensive and time-consuming processes, taking into account the additional devices’ integrations and time-consuming processes [69]. Manufacturing constraints are impeding the market launch of MF-based products, as is evidenced by their increasing relevance. This limitation has prompted the MF and analytical communities to investigate additive manufacturing (AM) as an alternative method for fabricating such devices, particularly now that 3D printers are more widely available [70].

AM is a procedure suitable for creating layer-by-layer (LbL) processes guided by 3D computer-aided design (CAD) modelling [71]. Considering its ability to construct complex forms, flexibility in design, and customization of a product, AM results in an inescapable threat to the traditional manufacturing method [72]. However, there are still limitations that need to be overcome before exploiting AM and 3D Printing (3DP) as scalable ready-to-use methods [73].

Concerns about inefficient or excessive extrusion processes, layer misalignment, and the need to make additional corrections after the production process are among the issues that most prevent AM from establishing itself as an industrial production technique [72]. Furthermore, some 3DP procedures may not always allow the LbL process to completely connect each layer, resulting in gaps that influence the mechanical properties of the produced product. Finally, while examining large-scale manufacturing processes, it is vital to highlight that the inability to create large-scale production in a single process, which must instead be produced at separate periods and then integrated together, hinders the AM from gaining a unique position [73]. Despite the above-mentioned issues, the 3DP approach is regarded as particularly effective when it is necessary to generate tiny objects with a high degree of complexity and the ability to quickly adjust the design. Furthermore, an in-depth examination of the printing settings may reveal a balance between process accuracy and time required. It is possible to actively work on the use of substrates that can be removed by modifying the object’s inclination, significantly reducing the quantity of raw material required, and the amount of energy used, paving the way for more sustainable processes compared to traditional methods [43,70,71]. Table 2 reports a practical comparison between 3D printing and the most commonly used traditional procedures for the production of MF devices.

Until recently, MF chips were manufactured using methods that necessitate a cleanroom environment and several post-production procedures. The novel feature of 3DP is the tightly integrated fabrication of an item from design software, which allows models to be quickly modified and repeated, resulting in an empirically informed recurrent design optimization loop. Finally, by combining design and materials, 3DP enables unprecedented levels of coordination and integration through the use of a fully or partially automated production system [75]. The most relevant MF-related 3DP facilities concern fused deposition modelling (FDM), light-triggered printing and inkjet 3D printing [75,76], which will be covered in this manuscript. A brief summary regarding the main 3D printing techniques used is presented in Table 3.

### 4.1. FDM

**Figure 3:**
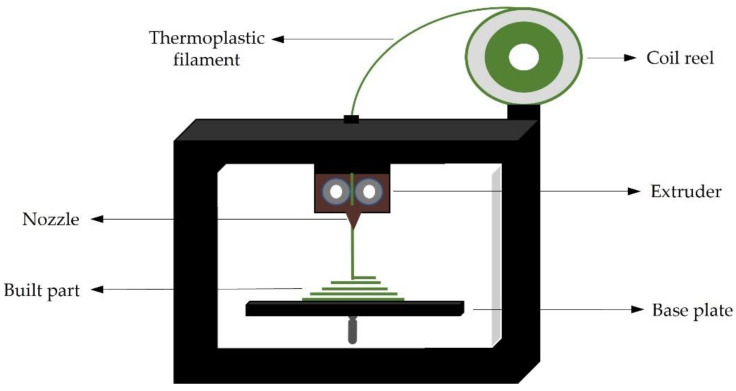
Simplified FDM 3DP schematic set-up.

FDM, also known as fused filament fabrication (FMM), is a 3DP method based on the use of a thermoplastic filament unrolled from a spool and pushed toward an extrusion head (including one or more extrusion nozzles) and drive wheels, which are required to keep the flow controlled [81]. The heater in the liquefier head is responsible for melting the filament to a semi-liquid state before extruding it through the nozzle to the printing area to manufacture the actual component [73]. The head may be manipulated horizontally and vertically by a numerical control system that follows a software-defined path [81]. The most crucial objective in this procedure is to fuse the successive layers before solidifying, since solidification before fusion might have a bigger influence on the other characteristics of the building portion [73,82]. A schematic set-up of a simple FDM printer is shown in Figure 3.

FDM provides features that make it suited for the manufacture of MF devices. These advantages include the printers’ reduced cost and accessibility, as well as their compatibility with a wide range of thermoplastic polymers, e.g., polylactic acid (PLA), polycarbonate (PC), polypropylene (PP), cyclic olefin copolymer (COC), polymethylmethacrylate (PMMA) thermoplastic polyurethane (TPU), and acrylonitrile butadiene styrene (ABS) [76]. Furthermore, FDM 3D printers may be set to work using several extrusion nozzles, realizing multi-material printing in a single process. This could represent a promising advantage, as they are able to merge, in a single step, useful materials with specific properties (e.g., conductivity, transparency, chemical resistance) [69,83]. Despite all the pros, commercially available 3D printers were considered unable to be used for MF device fabrication, due to their limited resolution, low optical transparency, high surface roughness, and challenges in manufacturing internal geometries smaller than 200 μm [41,76,84]. Nevertheless, some exploratory examples showing the promising possibilities of the FDM technique if used in combination with MF are listed below, demonstrating the great versatility of the technique.

Nelson et al., proved the FDM’s efficacy in the manufacturing of TPU-based MF devices. They generated microchannels (<100 μm) using a commercially accessible printer with a high degree of repeatability. In this work, it was demonstrated that TPU has far more potential in MF applications than PDMS, while remaining cost-effective ($0.01 per device) and providing very low production times (25 min). Taking the FDM technique into account, one of the limitations of molten polymer extrusion is the formation of voids, that could create reservoirs or points of fragility and leakage. By approaching the printing plate, the extrusion nuzzle was lowered to reduce the formation of these unwanted gaps. The extrusion lines were flattened as more polymer was deposited on the sides of the channels and the channel itself was narrowed, resulting in a print resolution of the smallest channels up to 40 μm. Working on the distance between the nozzle and the plate, it was also possible to increase the optical transparency of the printed product, which retains a certain roughness that represents one of the limitations of the technology. Furthermore, physical, and chemical stability studies have revealed that TPU-printed devices have greater elasticity and durability than PDMS, and could be used in high-pressure applications. In addition, the devices proved to be compatible with most organic solvents except chloroform and acetone. Furthermore, biocompatibility studies have shown that TPU does not induce cytotoxicity, demonstrating that it is a high-performance material suitable for a wide range of biological applications [85].

Intriguingly, da Bressan et al., obtained Au@Ag core-shell NPs using a PLA FDM-based MF device. In detail, the device included three inlets for reagent input and one outlet, and it was produced in less than two hours with 260 μm-sized serpentine channels. The internal device’s geometry enabled an optimal mixing between reagent phases, allowing the formation of core-shell NPs of approximately 23 nm size dimension. Despite the lower resistance to heat compared to ABS, PLA showed good transparency properties and reduced printing failure, being readily available and cost-effective (0.10 USA dollars per device) [86].

Klusák et al., employed a commercially available FDM printer to fabricate droplets MF devices. In this study, different microchips were designed and then printed using diverse materials. All MF devices had a crossflow geometry with various output channel widths (500, 700, 1000, and 1250 μm). Due to the FDM-related constraints, the device’s roughness depended on the printing technique, which was optimized to produce the best devices. Moreover, although the chips’ initial designs were squared, the printed ones resulted in circular cross channels. As a result, the 3D printed chips were able to generate highly homogenous microemulsions, and subsequently, a correlation between droplets size and fluid speed was studied to predict the performance of fully customized chips [87].

**Figure 4:**
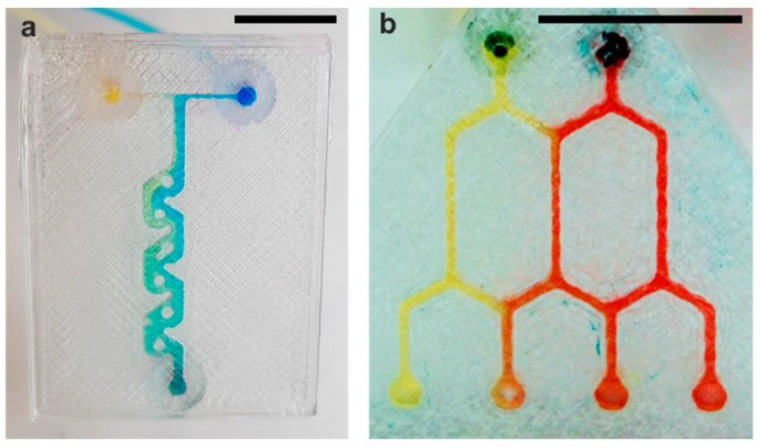
FDM printed microfluidic devices by Mader et al. [88]: (**a**) representation of Tesla-like micromixer; and (**b**) representation of cascade mixer.

Mader et al., described the creation of MF devices in polystyrene (PS), a polymer never before investigated in FDM. They established the viability of employing a thermoplastic polymer that is widely used in industry but is hardly utilized in AM. The authors achieved artefacts with channels with different geometries (Tesla-like micromixer and cascade mixer) of less than 600 μm within one hour (Figure 4). Furthermore, to achieve high transparency, a strategy was used that consisted of printing the microchannels directly onto PS substrate. UV/Vis analysis demonstrated that the devices had a transmission of over 50% in a range of 400–750 nm, showing good visual clarity. In addition, cell plate-like objects were printed, and cytocompatibility studies performed on them highlighted that PS has a huge potential in biological applications, as the industrially relevant materials could be exploited quickly and efficiently using the existing 3DP technology [88].

A final exemplary case may be found in a Quero et al. study, in which MF capillary electrophoresis (MCE) was manufactured with appropriately sized microchannels using an FDM printer. This work used different modified extrusion nozzles to achieve one-step multi-material printing. There are drawbacks to this method due to the small amount of molten material that adheres to the extrusion nozzle after printing a layer. This residual material may be lost on the next print layer as the nozzle moves, causing a problem when using materials with different properties. To address this issue, a device with a purge area positioned on the chip’s edge was designed. This innovation allowed for the printing of each layer without contamination by residual materials from the nozzle, which was removed in the purge area prior to the deposition of the new layer, allowing for the direct deposition of the conductive material that constituted the electrode. This evidence highlights the great versatility of the FDM technique, considering the multiple optimization possibilities, and its great future potential if applied to MF sensors [84].

### 4.2. Light-Triggered Printing

**Figure 5:**
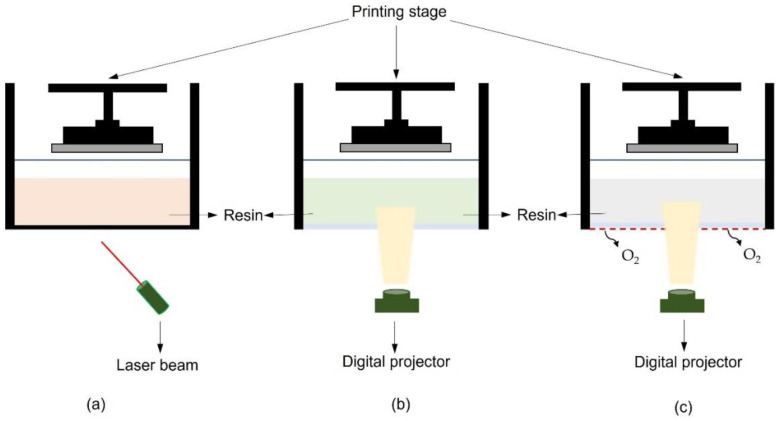
Schematic prototype of light source-triggered based printers: (**a**) simplified SLA printer; (**b**) simplified bottom-up DLP printer; and (**c**) simplified CLIP printer.

Photopolymerization-based 3DP processes are used to ensure the manufacture of high-resolution 3D objects. This method, which has gained popularity in recent years, is based on the employment of photocurable resins that polymerize in response to the impulse of a light source of specific wavelengths [89]. One of the initial attempts was based on stereolithographic SLA, a printing system in which a photosensitive material is polymerized according to the software’s instructions using a laser beam as a curing photoinitiator [90]. Digital light processing (DLP) and continuous liquid interface production (CLIP) represent a more recently explored advancement in UV-triggered based printing [89,91,92]. Although SLA and DLP employ the same working principle as rapid prototyping, in DLP the photoinitiator is represented by a digital projector [93]. The light path in DLP is direct, allowing for complete LbL photopolymerization at once, making this process quicker and slightly higher in resolution than similar SLA, in which the process is point-to-point laser-depended [79,94]. Despite several DLP printer machineries, all operate with the same fundamental set-up, which includes a building stage (or head), a resin container (vat), and a photoinitiator [26] (Figure 5).

Depending on the arrangement, DLP printers can be bottom-up or top-down equipped. Regarding the bottom-up shape, the moveable head is immersed inside the vat, allowing the resin to be irradiated from the underneath-placed UV-source [89]. This is possible due to the presence of a transparent vat bottom. A thin coating of resin is cured and remains attached to the head between the construction stage and the bottom of the vat. After the fixed polymerization time, the vertical movement of the head allows fresh resin to be put at the bottom of the vat for the next layer to be cured. In the top-down set-up, the photoinitiator is placed above and it radiates the resin in which the building stage is immersed [89]. After curing the first layer, the head travels lower, allowing fresh resin to be deposited on the plate’s surface for polymerizing the next layer. Both arrangements ensure the movement of the head in the vat, so that the amount of resin between the building stage and the photoinitiator is identical to the desired layer thickness [78,79].

To improve this technique, the novel CLIP system was recently studied. Although it is still less explored than SLA and DLP, they share the same UV-triggered photopolymerization mechanism. In this case, there is an oxygen-permeable window at the bottom of the resin reservoir that constantly supplies oxygen at the liquid interface. At the continuous liquid interface, an oxygen-enriched zone is created that is responsible for quenching the radical resin-curing process [92]. The photocurable precursor is cured by the light impulse derived from the digital projector underneath the reservoir [89]. The process allows the resin at the liquid interface to be constantly pushed into the gap via suction forces formed when the curing component is gradually moved away from the window. This approach enables a faster printing time, better surface properties of the printed items, and less roughness compared to DLP [92].

The implementation of SLA, DLP and CLIP printing is a feasible strategy that involves many advantages, e.g., cost-effectiveness, higher versatility and, in particular, the ability to print at lower temperatures with extreme visual clarity [89,95].

With DLP, achieving a performing printing resolution is possible by controlling the thickness of the cured layer. The cure depth of a specific resin is determined by the energy and light wavelength to which the resin is exposed. The tiniest features that may be printed are determined by the chemical molecular composition of the photocurable polymer, thus the smallest feature that can be generated is determined by the resin as well [96]. Hence the limited number of photocurable resins restricts these techniques’ fields of application [91]. With MF-based synthetic processes, one of the most crucial issues is the compatibility between the materials composing the device and the solvents to be utilized. When discussing the creation of MF devices by 3DP, it is essential to investigate a material’s printability, ability to build high-performance devices, and the required chemical compatibility [47].

Goralczyk et al., provided an interesting case study in this field, demonstrating the use of an SLA printer to produce basic MF chips made of perfluoropolyether dimethacrylate (PFPE-MA). Mechanical characteristics (up to 950 N load), thermal properties (up to 200 °C), and organic solvent resistance were also investigated. The chips were optically transparent, which is an important quality for an MF device. Three distinct geometries (serpentine mixer, Tesla mixer, and gradient mixer) were printed in less than 10 min, with 800 μm microchannels slicing thickness of 50 μm. The generated artefacts were evaluated for organic synthesis and found to be highly performant, demonstrating the suitability of this SLA as well as prospective uses for MF-based organic chemistry [97].

Moreno-Rivas et al. studied the printing via SLA of MF devices for cell cultures. The goal of this study was to demonstrate the feasibility of 3D techniques in the production of biocompatible devices. Interestingly, different photocurable resins were tested, demonstrating their optical and mechanical properties, including a study concerning the materials’ roughness. In addition, a method for ensuring cell adhesion to 3D printed substrates was reported, underlying the useful application of 3DP MF-based systems in biosensing [98].

**Figure 6:**
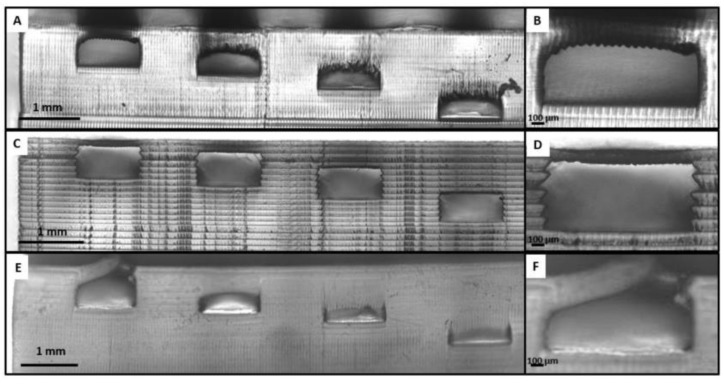
Images of the DLP 3D printed devices by Subirada et al. [99] using Detax (**A**,**B**), Asiga (**C**,**D**), and Keyprint (**E**,**F**) resins.

Subirada et al. used DLP to conduct a comparative study of the use of different resins in MF-based device 3DP (Figure 6). This research has shown that the one-step printing process has significant advantages over the traditional processes of part assembly. Indeed, eliminating the post-production phase reduces time, cost, and the possibility of errors. Furthermore, the presence of comparative studies on the properties of various printed materials is critical as it allows the selection of resin types based on the required properties [99].

The classical production processes of electrochemical MF devices (EMDs) require cleanroom environment, expensive equipment, and subsequent procedures to align and insert the electrodes within them. It is also important to test the compatibility of the various material and the lack of leakage to assess the robustness of the device. All these procedures result in expensive, high time-consuming outcomes [100]. Costa et al. demonstrated the feasibility of using commercially available SLA for building low-cost microchannels-based (100 × 200 μm) EMDs. In this study, it was possible to insert an electrode channel inside the main structure, avoiding alignment-related issues [100]. As reported in the work of Chen et al., the use of SLA-DLP 3DP to create flow-focusing MF devices used for liposome manufacturing was examined. In this example, a commercially available printer was employed to improve resins and printing conditions fabricating MF devices with dimensions of 200 μm. As a consequence, these devices were discovered to be suitable for producing lipid vesicles with adjustable characteristics less than 100 nm [101].

In another work, Shan et al., showed the rapid prototyping of MF devices exploiting a projection micro stereolithography (PμSL) 3D printer. The microchannels of the device were equipped with a three-layer layout, thus contributing to an increase in the total volumetric flow without affecting microchannel dimensions. In fact, this platform allowed for the rapid production of sized-controlled lipid nanosystems, ensuring a total flow rate (TFR) up to 474 mL min^−1^ [102].

Concerning the use of 3DP in the life sciences field, Tzivelekis et al., applied the knowledge of DLP-SLA for producing micro-chamber devices for polymerase chain reaction (PCR) molecular diagnostics. A two-phase process was implemented, firstly printing an open channel structure parallel to the projection plane without the use of supports. The removed platform was then covered with sacrificial paraffin wax, and a thin glass slide was placed to improve the quality of the subsequent printed layer. Finally, the second printing phase of the cap occurred. The obtained 3DP PCR chamber was cured with solvents in order to eliminate the wax and the excess of resin. This work, although still exploratory, showed the feasibility of the low-cost obtainment of life science micro-devices, opening the way to a novel and effective fast-production approach [103].

Liquid Crystal Display (DLC) is a light-triggered 3D printing technique based on the same working principle of DLP, using a precise UV-ray to induce resin polymerization. DLC is still less explored since there are relatively few compatible resins, yet it truly has several benefits, including high tensile strength and low shrinkage of the printed manufacts [104]. Recently, Weaver et al. explored the application of DLC in the production of novel MF devices showing different designs (namely Pug, Chihuahua, Retriever, Dachshund, Ridgeback, and Spaniel). The goal of this work was to combine the printed devices’ features with the MF conditions required to produce high-quality liposomal DDSs. In particular, it was demonstrated that the presence of a complex internal device geometry (e.g., Ridgeback and Spaniel) and the length of the main channel massively affected the repeatability and the polydispersity of the produced liposomes. Moreover, this study illustrated the pros and cons of using the LCD method applied to MF device fabrication. In-depth observations revealed that the DLC printed device offered a good resistance to working pressure up to TRF 7 mL/min, while it had limits circa the printing resolution at the smallest mixing angle (30°), causing clogging of the channels. Therefore, it is evident that the LCD technology offers important benefits in the field of AM, e.g., cost-effectiveness and faster production times compared to SLA, although additional research is required to address the problems related to printing resolution [105].

### 4.3. Inkjet 3D Printing

Inkjet 3D printing is a method that involves photopolymer-based and powder-based approaches. In this field, the most promising method associated with MF is photopolymer jetting, also known as the multiJet modelling (MJM) technique [80]. This a 3DP facility that exploits the jet of an acrylate–photopolymer upon a sacrificial support. Once the polymer is placed, each layer is cured by an UV light before next deposition. The great potential of this technique relies in the possibility of using several different materials in the same printing process without the need for additional assembly [75,76].

**Figure 7:**
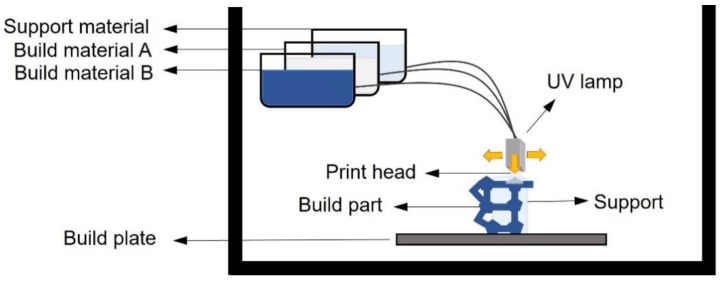
Schematization of MJM printing arrangement.

A simple schematization of MJM is shown in Figure 7. Compared to above-mentioned printing methods, e.g., FDM and SLA/DLP, material jetting is hampered in MF device production by the difficulty in creating empty spaces or voids inside microchannels [80]. Despite the difficulties, some examples of attempts to improve material jetting and making it more suited for MF device fabrication have been found in the literature and are reported here.

The main feature that raised the significance of MJM printing approach concern the possibility to print and integrate materials with distinct properties. An interesting step about this feasibility has been conducted by Jin et al., that soft and hard materials were printed using MJM to produce a MF droplet generator and a pneumatic control unit (PCU). The great novelty of this work relies in the possibility to print a channel with a rubber-like material and a rigid structure in the same monolithically device. This allowed to generate droplet in a controlled manner inside the flexible microchannel with the use of air pressure. In this case an integrated device was produced using MJM, in one-step, without the need of complex post-production processes. Compared to PDMS, TangoPlus material is softer, as it allows more thicker flexible channels, providing robustness and stability of the device. Thus, the beneficial potential of multimaterial printing was demonstrated [106]. As previously stated [80], the incapacity to print tortuous microchannels or gaps without the use of a sacrificial support renders material jetting unsuitable for the manufacture of MF devices as the removal of support material out of microchannels is time consuming or nearly impossible.

Castiaux et al., tried to avoid these limitations, showing two novel methods to print devices without the need of supports using a material jetting technique. The two approaches consisted in minimal to no post-processing, allowing the fabrication of devices with intricate geometries (serpentine and Y-mixer). One protocol involved printing open microchannels, which were then filled with a liquid support solution; the other procedure implicated the use of a polycarbonate membrane as a solid support. In both cases, after placement of the support to allow the photopolymer to be cured, a second printing step was conducted to ensure the cover and closure of microchannels. This novelty assured better prints compared to traditional multi-jet modelling, enabling the production of enclosed MF channels. Eliminating the sacrificial supports leads to a consistent advantage as the process is less time-consuming and avoids the removal of embedded material that could clog the channels [107].

Enders et al., used a high-definition MJM printer to produce several different micromixers suitable for integration within MF devices. Five mixer geometries (T-mixer, Caterpillar mixer, enhanced Caterpillar mixer, and HC-mixer) were printed scaling the dimensions according to cell suspension use. Comparative studies between the diverse mixers’ capacity, showed that the Caterpillar mixer and HC-mixer were the best performing, enabling a complete mixing of fluids in less time. Moreover, it was demonstrated that these devices were suitable for performing mammalian cell DNA transfection, opening the way to widespread application of the MJM printer for biological application purposes [108].

**Figure 8:**
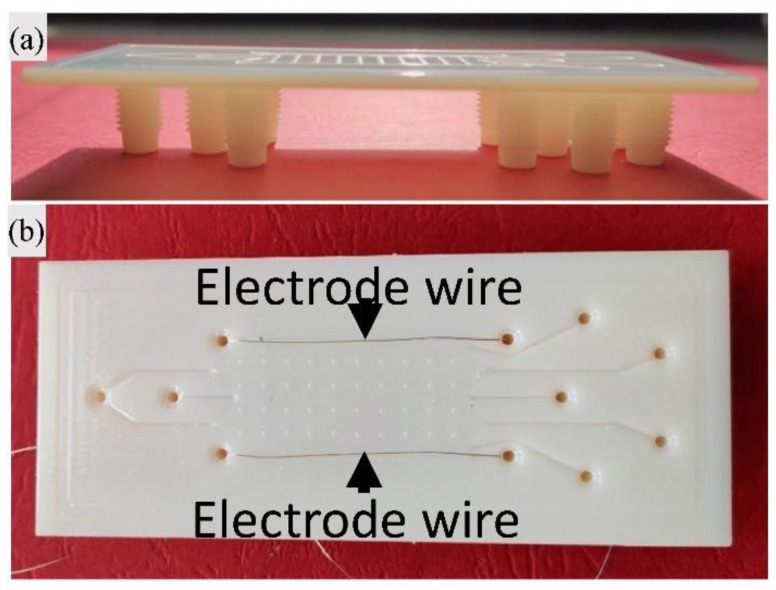
Images of μFFE device produced by Barbaresco et al. [109]: (**a**) image of 3D-printed device; and (**b**) image of 3D-printed μFFE device after the electrode insertion.

Subsequently, Barbaresco et al., produced micro free-flow electrophoresis (μFFE) devices to achieve rapid micro (M) and NP testing. The fabrication process of these micro systems, useful for LOC application, was conducted using an inkjet 3DP. In this research two different “glossy” and “matte” features of the commercially available resin were applied. As a result, the “glossy” printed μFFE showed higher accuracy and resolution compared to the FDM-printed ones (Figure 8) [109]. When FFE systems were used, they were able to differentiate M/NPs based on their size/charge ratio, demonstrating the strategy’s feasibility. The ability to fully customize chips provides numerous benefits, including device optimization through a “try-and-error” approach, the ability to add multiple inlet/outlet ports, and improved accuracy when compared to other printing strategies. The chips were printed with a 5% accuracy of the CAD designs, demonstrating that the MJM printing technique is suitable for the production of low-cost, effective μFFE systems [109].

## 5. The Synergy of MF and 3DP

It is now more important than ever to accelerate research into new production strategies in order to produce novel nanoformulations in a controlled and continuous manner suitable for industrial scale-up. Despite some production challenges, time-consuming processes, and prohibitive costs, the use of NM in life science should become a concrete reality in the coming years. Following an examination of the potential of MF and 3DP as semi-independent techniques, it is critical to consider the extraordinary potential that could be explored if the two techniques were used concurrently.

Interestingly, Chang et al., used a low-cost commercial FDM system to print MF devices for the synthesis of anticancer nanoformulations. Each device had a passive micromixing structure with a zig-zag geometry. After the PLA devices were printed, they were used to create organic metal NPs made of copper/Disulfiram complexes [Cu(DDC)_2_MONs] coated with bovine serum albumin. The optimized formulation was characterized by narrow size (<100 μm) and highly mono-dispersed. Compared to the classical “vortexing method”, the MF-based approach ensured the production of 240 mL of the formulation per hour using the 3D-printed device, providing an unprecedented advantage in terms of time consumption. Furthermore, these innovative MF nanosystems have been tested on breast cancer models, and were shown to be effective on in vitro tumor inhibition [110].

Kara et al., demonstrated the promising results of combining MF and 3DP, and MF-based devices were successfully manufactured using both FDM and SLA printers. Both devices were designed with two inlets and one output for the outcome; they showed no porosity or leakage, demonstrating the high performance of both FDM and SLA techniques, and a lack of errors in the layers deposition and polymerization, respectively. Devices printed using the SLA printer showed smoother channels, since the printing strategy is better-performing in terms of resolution and surface roughness properties. As a result, these devices were used to develop Nifedipine-loaded polymeric nanosystems smaller than 100 nm. When compared to traditional fabrication methods, FDM and SLA saved time and costs. Interestingly, the NPs produced using 3D-printed devices exhibited properties similar to those produced using the traditional solvent-evaporation method. Despite the promising data obtained when applying both MF devices, this comparative study highlighted the superior validity of SLA as a printing technique. In fact, the SLA-based device provided enhanced mixing properties in the fabrication of the best-performing polymeric NPs [111].

Recently, an FDM 3D printer was used by Tiboni et al., to produce PP MF devices with two different internal geometries. The application of AM allowed for the achievement of featured chips with, respectively, “zigzag bas-relief” (Z chip) and “split-and-recombine (SAR)” (C chip) channel shapes. The effective dimensions of the channels were evaluated with a digital microscope and were shown to accord with the CAD project and had good printing resolutions. Computational fluid-dynamic simulations demonstrated the improved mixing abilities of both devices. The use of PP as a material resulted in reusable, robust, flexible devices that were inert to organic solvents. These chips were used for polymeric NPs and liposomes production; it was found that both polymeric and lipidic concentrations affected the quality of the outcome. The size of the liposomes was heavily influenced by the chip design, while for polymeric NPs, it was more important that TFR was used [112].

Intriguingly, Sommonte et al. exploited the same Z chip [112] to produce enzyme-loaded solid lipid nanoparticles (SLNs) via an MF-based strategy. The presence of bas-reliefs within the main channel allowed for passive chaotic advection to improve the degree of mixing of the two fluid phases while achieving a TFR of up to 30 mL/min. This research was based on a comparison of SLNs made using the conventional approach and those that were MF-based. It is interesting to note that MF manufacturing of lipidic NPs was found to achieve better results than the traditional production method. The MF-based enzyme-loaded SLNs that were produced showed narrower size minimal polydispersion and were extremely repeatable. In addition, the higher encapsulation efficiency confirmed that there was no interaction of the encapsulated drug with the device material (PP); also, the released enzyme was shown to be active on its biological substrate, ensuring that MF production preserved its activity.

This study presents the proof-of-concept that combining 3DP and MF enables faster and more cost-effective novel DDS production, solving the reproducibility issues related to traditional methods. Moreover, it was shown that the activity of the encapsulated enzyme into SLNs was preserved, opening the way to a possible application of biomolecules-based nanoformulations in NM, exploiting the synergy of 3DP and MF [113].

Furthermore, Drishya et al., performed the production of a simple T-junction MF chip with an SLA printer. This device was demonstrated useful in the production of Resveratrol- and Curcumin-loaded anticancer emulsion. The application of MF enabled the production of stable smaller droplets with higher encapsulation efficacy, 65.11% and 58.40% for Curcumin and Resveratrol respectively, and low polydispersity compared to syringe pump and hand injection traditional methods. Moreover, this study demonstrated the importance of strict control over the flow rates, as it assured the preparation of stable emulsions and smaller droplets, resulting in enhanced stability [114].

Vasilescu et al., used the DLP technique to design and print MF devices with complex microchannel geometries. Each device was characterized by micromixer design with threaded microchannels to enhance convective diffusion. Additive manufacturing was the key point in producing threads that promoted the fluids’ higher contact area, enhancing the mixing index (MI). In fact, both devices were found to achieve circa 100% of MI. This geometry was useful in creating a highly efficient micromixer capable of inducing conjugation between AuNPs, PS-NPs, and the antibody (Ab). The high MI induced by 3D-printed chips was able to reduce the time required to complete the reaction compared to batch scale incubation, demonstrating a significant improvement in the use of 3D-printed MF-based systems. As a result, this has had a significant impact in biomedical fields such as theragnostic and biosensing [115].

A useful tool to produce iron oxide core chitosan NPs, Aşık at. al., was produced by a MJM printer.

Two different MF devices were produced by flow-focusing junction geometry; the first had a straight channel, while the other was characterized by hurdles within it. Using the two different chips, it was found that there were important differences in NP size based on the fluids rates tested. Implementing the geometry micromixing with the use of hurdles resulted in rounder and less angular nanosized NPs compared to those produced using the straight microchannel, demonstrating the feasibility of the improved technique. As a result, this study demonstrated the significance of device geometry in the production of NPs available for various applications in NM, emphasizing the benefits of 3D printing as a versatile approach which allows for fast customization based on required needs [116].

In a recent study, Chen et al., implemented a never-explored interconnected MF device for multi-drug combinations for anticancer application. A one-step MJM technique was exploited to fabricate a high-throughput device with four inlets and thirty-six outlets displayed in four layers. The internal geometry was based on a multi-layer tree-shaped branch unit. An SAR mixing process was allowed by the presence of interconnected network channels. Thirty-six concentrations of antitumor combinations were created as proof of the effectiveness of this new synthetic and screening strategy, and their activity was tested on human lung cancer cell lines. The outcomes have exceeded all expectations. This intricately designed device has great potential for use in the synthesis of new compounds, as well as for the analytical screening of various multi-drug combinations. Since this strategy allowed data to be obtained in less time than traditional NM research, it represents a potential tool to completely change the approach to searching for novel therapeutic treatments [117].

**Figure 9:**
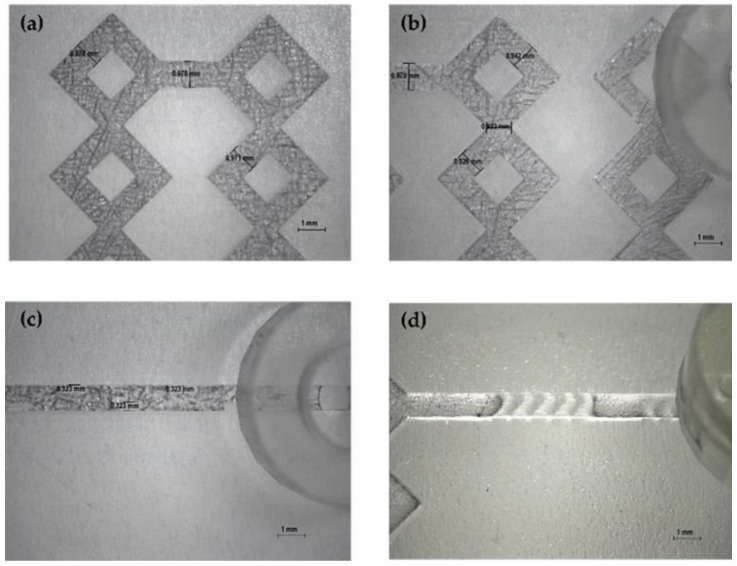
Optical microscope images of diamond-shaped devices produced by Sommonte et al. [118]: (**a**,**b**) images of the circumvolved internal geometry; (**c**) image of the main channel with wedges inside; and (**d**) image of the main channel with modified herringbone structure inside.

Finally, Sommonte et al. established the viability of the synergy between 3DP and MF in a recently published paper. CAD software was utilized to create *in-house* diamond-shaped devices suitable for the manufacture of liposomes carrying lysozyme as a model drug. Four innovative chips were purposefully developed with an interconnected internal path to increase mixing between the organic and aqueous phases, and two more devices, namely, modified herringbone and wedges, were generated with obstacles inside the main channel to exploit the chaotic advection phenomenon. The devices were printed using a high-performance DLP 3DP printer, resulting in extremely high-resolution chips (Figure 9) that were tested to optimize the experimental conditions for producing MF-based liposomes. Using an *in-house* facility, monodisperse, narrow-sized, lysozyme-loaded PEGylated liposomes with an ideal size (143 ± 8 nm) and PDI (0.15 ± 0.01) were synthesized. Moreover, the better-performing formulations were subjected to a stability study and an in vitro release analysis to assess their consistency. In this case, the excellent visual clarity of the DLP-printed devices were demonstrated, thus the name “diamond”, and the significant benefit and possibility of customizing MF devices in a short period of time based on requirements was also demonstrated [118].

**Figure 10:**
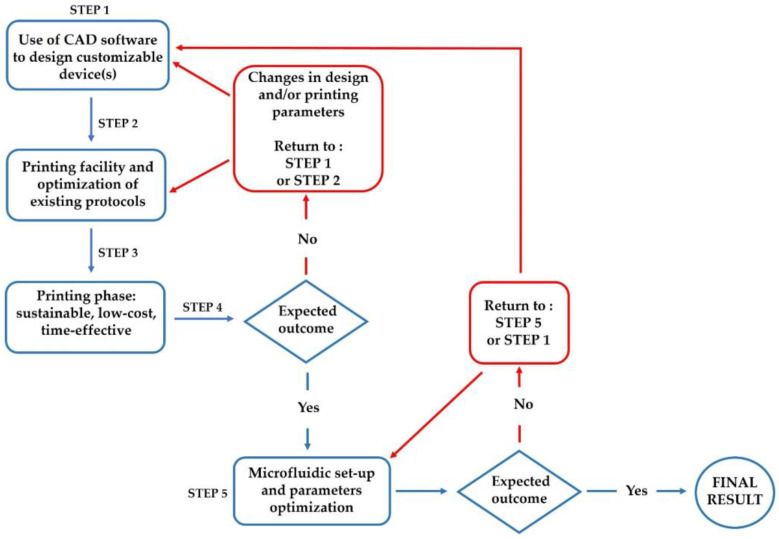
Schematic flowchart regarding the combination of 3DP and MF. The diagram is an example of an event sequence in a synergistic protocol. As illustrated, if the printed product does not conform to the expected one, it is feasible to swiftly adjust the design (returning to **STEP 1**) or the printing parameters (returning to **STEP 2**). Similarly, if the MF application of the device does not produce the expected result, the MF parameters can be adjusted (returning to **STEP5**) or the object’s geometry can be changed (returning to **STEP 1**). This may be completed quickly, and it is entirely adjustable to the needs and desired outcome.

The several examples covered in this session demonstrate that the field of MF and 3DP offers a wide variety of benefits that have yet to be completely explored (Figure 10). While it is now widely recognized that both approaches constitute significant advances if applied to the biological sciences, the full potential of their synergy remains relatively unexplored. The following are some major points that summarize the combination’s advantages:

- The possibility to create diverse and distinct MF devices individually, examining from case to case the numerous geometries that may be entirely tailored in line with the various desired results, using readily available CAD applications [22,70];
- The ability to select the material to be printed, given the wide range of options available, depending on the printer’s specifications and the characteristics desired, such as transparency, resistance to organic solvents, thermostability, and compatibility of surface interactions with substances to be encapsulated within nanoformulations [22];
- The full customization of designs about 3D printed MF devices. With the addition of previously unexplored components, new geometries may be examined. The devices are low-cost, reusable, and more sustainable than those made industrially and subjected to post-processing methods once optimized in the production phase and with reduced energy and material waste [43,112];
- The developed devices may be evaluated for all the formulations that are predicted to be generated in a relatively short time, since they are ready to use once manufactured, and this technique therefore provides for the rapid completion of experimental and comparative research [117].

## 6. Expert Opinion and Future Directions

Every day, scientific research yields new discoveries with highly practical applications due to the plethora of biotechnological, chemical, engineering, and computer techniques now available [119,120]. However, these previously unseen discoveries are frequently hampered in their applicability due to a lack of appropriate techniques for their development. The area of NM, which is now more relevant than ever, provides constant insights into the extent to which the use of new formulations is hindered by the impossibility of safe, fast, large-scale production [121]. In terms of data reported in the literature, the last few years have seen an unprecedented surge in the fields of MF and AM [24,122]. Because they were designed to be scalable techniques that could be transferred to the industrial sector, both techniques have distorted the scientific field. Using the COVID-19 pandemic as an example, it suddenly became clear that scientific research applied to public health urgently requires technologies capable of responding promptly to emergency health conditions [11]. A good example is the conversion of many companies that dealt with 3D printing in various industrial fields, which quickly converted to the production of readily available medical devices for the fight against the pandemic [123]. In addition, one of the most revolutionary innovations that the combination of MF and 3DP can bring is the ability to concentrate all laboratory operations, from the simplest to the most complex, in a small layout that can be self-produced in a short time. Until recently, it would have been unthinkable to obtain MF devices for $0.10 in less than two hours, now it is a viable reality [85,86].

The novelty of MF and 3DP is that, while both techniques have limitations, they are completely customizable. The personalization of the technique should not be interpreted solely in terms of the type of object to be printed, but rather as encompassing a broader range of possibilities. Engineering an MF setup or a new AM system, optimizing operating parameters, and testing previously unexplored approaches are illustrative examples of how tools alone do not constitute a facility, but it is the operator who studies them who can discover new ways to exploit their applications [124]. All the examples in the preceding sections were chosen to demonstrate how research aimed at exploring alternative methods can provide unimaginable advantages over traditional methods. Furthermore, research is evolving toward a more green and sustainable approach in order to have the least possible environmental impact. Both MF and 3D printing are highly sustainable methodologies as they reduce the consumption of energy and raw materials by definition [43]. Given the scientific trends of recent years, future perspectives must include the continuous development of these two techniques in combination to realize an industrial scale-up, exploring all the possibilities of large-scale application.

## 7. Conclusions

The goal of this review was to highlight the benefits of using MF and 3DP individually, as well as to emphasize the innovations that could be made when using these two techniques together in the field of life science. The concept of synergy assumes that such a combination can overcome many of the challenges that still exist in translating NM into clinical applications. Although the two techniques are well-established, the use of synergy is still in its early stages. The ability to combine MF and 3DP is one of the most desirable innovations of the coming years, so research in this area is thriving. The advantages of the two techniques alone would be greatly expanded, paving the way for faster, more sustainable scaling-up results.

## Figures and Tables

**Table 1:** A brief overview of traditional MF device manufacturing methods based on construction material.

Material	Fabrication Method	Refs.
Glass	Laminate manufacturing Wet etching/Dry etching Micromilling Microgrinding	[46,48,64]
Silicon and polymeric materials	Lithography Laser ablation Hot embossing Inject moulding Soft lithography	[44,46,49,65]
Metals	Laser ablation Lithography	[65]
Paper	Paper cutting Ink plotting Wax/inkjet/laser printing Plasma etching Photolithography	[66,67,68]
Hydrogels	Micromoulding Sacrificial template replication Photo-patterning	[56]

**Table 2:** Comparison regarding pros and cons of using traditional and 3D printing methods in MF device manufacturing.

Material	Production Method	Advantages	Disadvantages	Refs.
Glass	Laminate manufacturing	Easy to use, scalable	Misalignment of layers, air bubble, possibility of leakage, time-consuming	[64]
	Wet/Dry etching	Fast, precise	Expensive instrumentation, low etching rate	[48]
	Mechanical processes	Crack-free surfaces	Low precision, need of controlled environment	[48,74]
	3D printing	Time-effective, crack-free processes	Need of heated chamber to prevent thermal shock	[74]
Polymeric materials	Soft lithography	Suitable for most materials, scalable, easy to use	Easy to deform, Low repeatability	[49]
	Hot embossing and imprinting	Rapid, high resolution and precision	High cost	[49]
	Laser ablation	Fast	High cost, elevate roughness of surfaces	[49]
	Lithography	High resolution, good reliability	High cost, no scalable procedures	[49]
	3D printing	Customizable features, low-cost, time-effective, printing on-demand	Not suitable for all materials, resolution issues	[46,49]
Hydrogel	Micromoulding	Controlled microstructures	Stress damages during demoulding	[56,62]
	Photo-patterning	Fast, high resolution and repeatability	Restricted to photo-sensitive hydrogels	[56]
	3D printing	Time-effective, controlled microstructures, lower cost	Restrictions related to mechanical properties	[62,63]

**Table 3:** Short overview about main 3DP techniques advantages and drawbacks.

3D Printing Technique	Material Type	Pros	Cons	Refs.
FDM	Thermoplastic filament	Rapid prototyping, low-cost, no post-production processes, easy to use	Mechanical drawbacks (air gap, layers misalignment), poor surface properties, low optical transparency	[71,72,77]
Light- triggered printing	UV curable resin	Highest accuracy, visual clarity, high mechanical properties, smooth surface	Post-production requirement to remove uncured resin, less cost-effectiveness material, delamination process	[71,72,78,79]
Inkjet printing	UV curable acrylic	High speed, possibility to use different material in the same print	Lack of adhesion between layers, coarse resolution, post-production removal of support, difficulty to fabricate “voids”	[72,75,80]

## Data Availability

Data is contained within the article.
